# Ten tips on immunosuppression in primary membranous nephropathy

**DOI:** 10.1093/ckj/sfae129

**Published:** 2024-04-29

**Authors:** Hernando Trujillo, Fernando Caravaca-Fontán, Manuel Praga

**Affiliations:** Department of Nephrology, Hospital Universitario, 12 de Octubre, Madrid, Spain; Department of Nephrology, Hospital Universitario, 12 de Octubre, Madrid, Spain; Instituto de Investigación Hospital, 12 de Octubre (i+12), Madrid, Spain; Department of Medicine, Universidad Complutense de Madrid, Madrid, Spain

**Keywords:** anti-PLA2R antibodies, cyclophosphamide, immunosuppressive therapy, membranous nephropathy, rituximab

## Abstract

Membranous nephropathy (MN) management poses challenges, particularly in selecting appropriate immunosuppressive treatments (IST) and monitoring disease progression and complications. This article highlights 10 key tips for the management of primary MN based on current evidence and clinical experience. First, we advise against prescribing IST to patients without nephrotic syndrome (NS), emphasizing the need for close monitoring of disease progression. Second, we recommend initiating IST in patients with persistent NS or declining kidney function. Third, we suggest prescribing rituximab (RTX) or RTX combined with calcineurin inhibitors in medium-risk patients. Fourth, we propose cyclophosphamide-based immunosuppression for high-risk patients. Fifth, we discourage the use of glucocorticoid monotherapy or mycophenolate mofetil as initial treatments. Sixth, we underscore the importance of preventing infectious complications in patients receiving IST. Seventh, we emphasize the need for personalized monitoring of IST by closely measuring kidney function, proteinuria, serum albumin and anti-M-type phospholipase A2 receptor levels. Eighth, we recommend a stepwise approach in the treatment of resistant disease. Ninth, we advise adjusting treatment for relapses based on individual risk profiles. Finally, we caution about the potential recurrence of MN after kidney transplantation and suggest appropriate monitoring and treatment strategies for post-transplantation MN. These tips provide comprehensive guidance for clinicians managing MN, aiming to optimize patient outcomes and minimize complications.

## TIP 1. DO NOT PRESCRIBE IMMUNOSUPPRESSIVE TREATMENT (IST) TO PATIENTS WITHOUT NEPHROTIC SYNDROME (NS)

Available data show that a high percentage of membranous nephropathy (MN) cases with non-nephrotic proteinuria (up to 40%) do not progress to nephrotic syndrome (NS) [1]. These cases should be closely followed without starting IST and watching the trend of the disease towards spontaneous complete remission, maintenance of non-nephrotic proteinuria or development of full NS.

Supporting a conservative attitude in these patients is a better tolerance to the amount of proteinuria in MN compared with other glomerular diseases such as immunoglobulin A nephropathy [2]. On the other hand, the number of non-immunosuppressive antiproteinuric drugs has increased in recent years. Two large randomized trials [DAPA-CKD (NCT03036150) and EMPA-KIDNEY (NCT03594110)] found that dapagliflozin and empagliflozin decrease the risk of CKD progression by 32% regardless of the presence of diabetes [3–5]. Both trials enrolled a combined total of 136 patients with MN [6]. In a recent large retrospective cohort study, the use of sodium–glucose co-transporter 2 inhibitors (SGLT2is) was associated with a proteinuria reduction of 48% after 1 year under SGLT2i in a diverse subset of primary and secondary glomerular diseases, including 89 patients with MN [7]. Results were similar irrespective of the underlying disease. Therefore, the combination of SGLT2i and renin–angiotensin–aldosterone system (RAAS) blockade can substantially reduce the amount of proteinuria in MN patients without complete NS.

An important limitation of the above statements is that the main studies on spontaneous remission of MN and on the natural history of MN with non-nephrotic proteinuria were published before the discovery of anti-M-type phospholipase A2 receptor (PLA2R) antibodies and other MN-associated antigens [1, 8–10]. Initial anti-PLA2R titres, and especially its evolution, should be carefully monitored in these patients [11–13].

## TIP 2. PRESCRIBE IST TO THOSE PATIENTS IN WHOM NS DOES NOT IMPROVE AFTER AN OBSERVATION PERIOD AND IN THOSE WITH DECLINING KIDNEY FUNCTION OR SERIOUS COMPLICATIONS OF NS

Spontaneous remission (SR) is relatively frequent in MN (≈30% of cases), especially in those with lower proteinuria [8, 10, 14, 15]. As well as the amount of proteinuria, other predictors of a higher probability of SR include female sex, baseline kidney function, treatment with RAAS blockers and a decrease in proteinuria >50% in the first year. SR usually develops within 24 months since MN diagnosis and is generally associated with good long-term prognosis and low recurrence rate [10]. Thus an observation period is highly recommended before IST is started.

In anti-PLA2R-positive cases, both baseline titres and the trajectory of the autoantibodies are crucial to determine the prognosis and the need for IST. Immunologic remission has been associated with clinical remission and higher antibody titres have been linked to lower rates of remission and worse kidney survival [16–18]. A 50% decrease in anti-PLA2R precedes a similar reduction in proteinuria by ≈10 months [18].

The length of the observation period should be individualized, but in general, initiation of IST is recommended if there is no trend towards improvement in disease activity (decreasing proteinuria/anti-PLA2R titres, increasing serum albumin) after 6–9 months. In contrast, we strongly advise initiating immunosuppression without delay in patients with deteriorating kidney function not attributable to other causes (e.g. diuretics overuse and high doses of RAAS blockers in the presence of low blood pressure) [19, 20]. Also, IST should be started in patients with massive oedema resistant to diuretics and/or severe complications of NS (such as pulmonary thromboembolism). Similarly, it seems reasonable not to wait in patients with massive proteinuria (>10–12 g/24 h), severe hypoalbuminemia (<2 g/dl) and very high anti-PLA2R titres (>275 RU/ml) [18], as the probability of SR in these cases is low.

## TIP 3. PRESCRIBE RITUXIMAB (RTX) OR RTX COMBINED WITH CALCINEURIN INHIBITORS (CNIS) IN PATIENTS CATEGORIZED AS MEDIUM RISK FOR PROGRESSIVE LOSS OF KIDNEY FUNCTION

**Figure 1:**
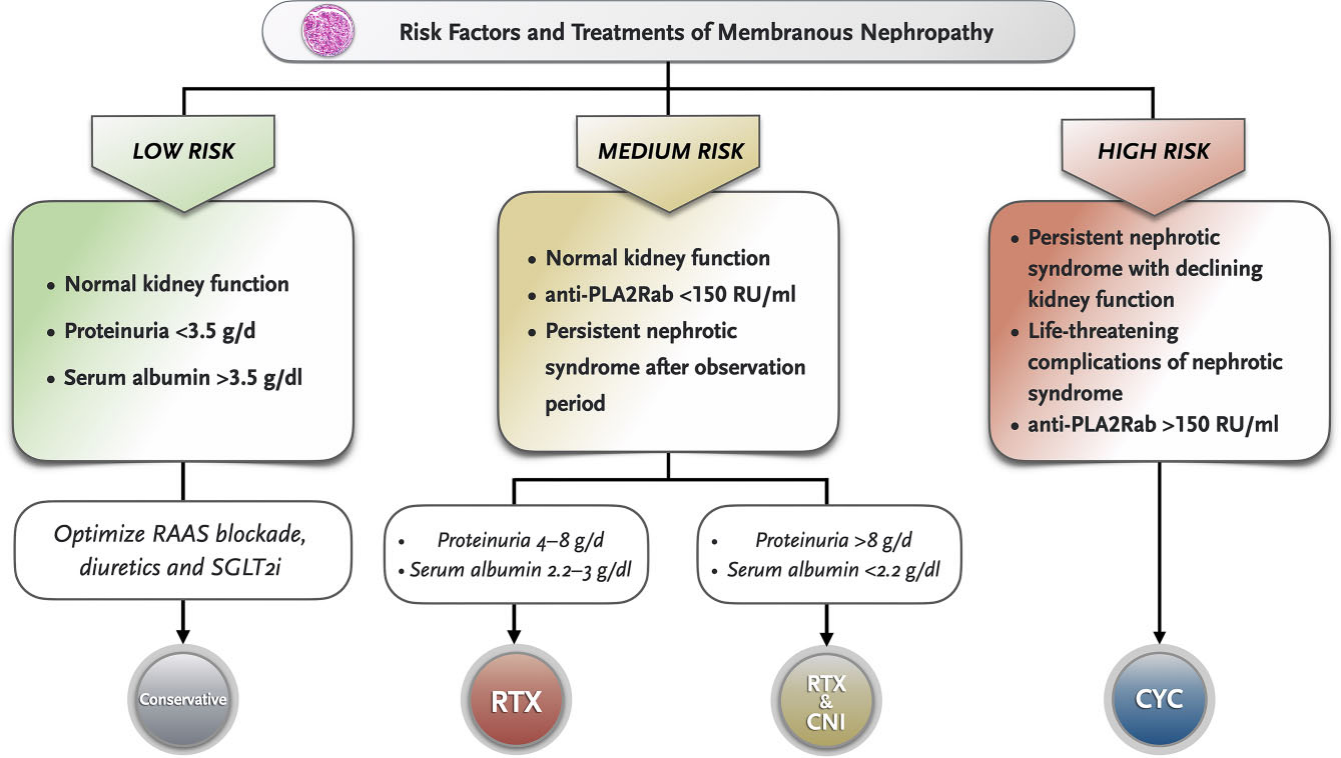
Risk factors for kidney disease progression in MN and proposed therapeutic approach. aPLA2Rab: anti-phospholipase A2 receptor antibody; RAS: renin–angiotensin system; RTX: rituximab.

We advise categorizing patients according to their risk factors for progressive loss of kidney function (Fig. 1) and prescribing RTX or RTX combined with CNIs in medium-risk patients. Observational studies have shown a beneficial effect of monotherapy with RTX in MN [21, 22] and this has been confirmed by several well-known prospective trials [23–25] (Table 1).

**Table 1:** Percentage of patients achieving CR or PR in recent randomized controlled trials comparing different treatment schemes.

	CRs + PRs, %		
Therapeutic regimen (trial)	6 months	12 months	24 months
RTX (GEMRITUX)	35	–	65
RTX (MENTOR)	35	60	60
RTX (RY-CYCLO)	51	62	85
Cyclosporine (MENTOR)	49	52	20
Tacrolimus + corticosteroids (Ramachandran *et al.* [29])	74	71	43
Tacrolimus + RTX (STARMEN)	44	51	58
GC–CYC (Ramachandran *et al.* [30])	60	77	80
GC–CYC (STARMEN)	74	79	84
GC–CYC (RY-CYCLO)	65	73	81

Treatment with CNI (tacrolimus, cyclosporine) has also proven to be effective [26–30] (Table 1), but the major drawback of CNI monotherapy is the high rate of relapse after withdrawal. Risk factors for relapse include high residual proteinuria after partial remission (PR) is attained and rapid withdrawal of the drug [28]; therefore, it is advisable to perform a very slow tapering (9–18 months, depending on the amount of residual proteinuria) [28, 31] or to add RTX at the beginning of CNI withdrawal since the probability of relapse may be significantly reduced [32, 33].

**Figure 2:**
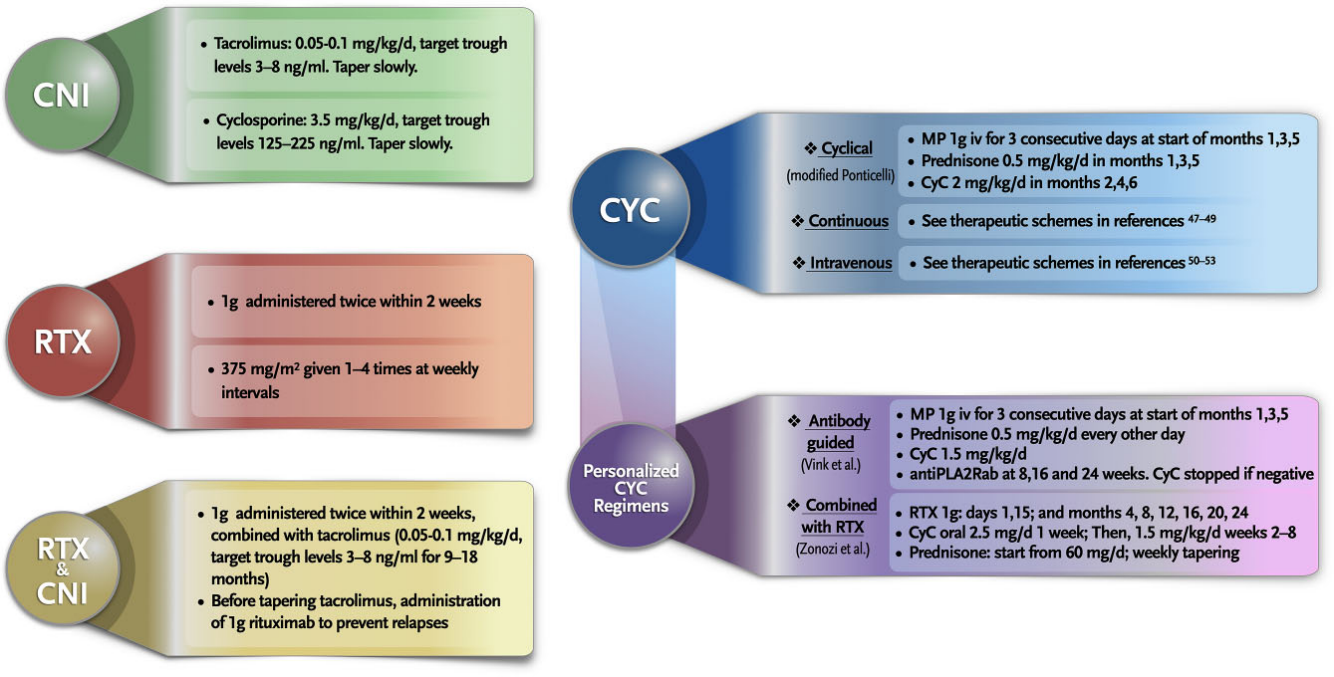
Treatment regimens for MN.

The proven efficacy of RTX and a better tolerance profile as compared with cyclophosphamide (CYC)-based therapeutic regimens has led to the use of RTX as first-line therapy in most patients without severe risk factors. The ideal RTX dose is unclear (Fig. 2); some studies have found that relatively low doses of RTX may result in worse outcomes than conventional doses [34].

One handicap of RTX is that the therapeutic effect is relatively slow and more than half of the patients persist with NS after 6 months. The latter can be countered by adding a CNI, with the advantage of a faster antiproteinuric effect, especially in cases with elevated proteinuria or severe hypoalbuminemia [33, 35] (Fig. 1). More studies are needed to clarify the most effective regimens of RTX in monotherapy or combined with CNI.

## TIP 4. PRESCRIBE CYC-BASED IMMUNOSUPPRESSION IN PATIENTS AT HIGH RISK FOR DISEASE PROGRESSION

The efficacy of alkylating agents alternating with glucocorticoids (GCs) (i.e. the modified Ponticelli regimen) has been demonstrated in several prospective studies [36–40] and extended follow-up showed that alkylating agents decrease the risk of long-term kidney failure [41]. Nevertheless, the use of CYC is currently restricted to high-risk patients (Fig. 1) due to potential toxicity and the risk of serious adverse effects. Since CYC is associated with fewer side effects than chlorambucil [39], the latter has been almost completely relegated.

Few controlled studies have directly compared the Ponticelli regimen with other therapeutic alternatives [25, 29, 30, 33], but CYC-based regimens have generally been demonstrated to induce a higher percentage of remissions (Table 1). A randomized controlled study demonstrated the superiority of cyclical chlorambucil plus GC over cyclosporine or supportive care in patients with declining kidney function [19]. Likewise, observational studies have shown that CYC could be more effective than RTX in patients with high anti-PLA2R titres (>150 RU/ml) [42], although response to RTX in such cases has also been reported [43]. Based on the above-mentioned data, we suggest a preferential use of CY-based regimens in high-risk patients (Fig. 1).

The safety profile (risk of sepsis, malignancy, myelotoxicity and infertility, among others) of CYC is closely related to the total cumulative dose [44, 45]. The modified Ponticelli regimen usually involves a cumulative dose of 10 g, but other oral CYC protocols may entail significantly higher doses [46]. Different therapeutic CYC-based regimens have been proposed [47–53] (Fig. 2).

The STARMEN trial (NCT01955187) found that in the arm of cyclical CYC-GC, proteinuria had been significantly reduced and there was already a high rate of immunologic remissions after only 3 months of treatment (cumulative CYC dose of 3.7 ± 1.1 g) (Table 2), suggesting that shorter courses may be as effective as the classically prescribed 6-month schedule. Also, recent studies advocate that the duration and cumulative dose of CYC can be reduced and individualized according to the patient's clinical and immunological response, either with cyclical oral CYC plus GC according to longitudinal anti-PLA2R measurements (at 8, 16 and 24 weeks) [54] or with the use of pulse intravenous CYC instead of oral administration [50, 52, 53] (Fig. 2). Prospective studies comparing the modified Ponticelli regimen with lower-dose CYC schemes are needed.

**Table 2:** Monthly evolution of patients treated with corticosteroid–cyclophosphamide in the STARMEN trial.

Characteristics	Baseline	Month 1	Month 2	Month 3	Month 4	Month 5	Month 6
Proteinuria (g/24 h), median (IQR)	7.4 (4.8–11.3)	4.4 (2.8–7.3)	3.8 (1.7–5.9)	3 (1.8–5.9)	1.8 (1–4.6)	2.1 (1.3–4.9)	1.2 (0.6–3.4)
Change from baseline (%), median (IQR)	–	−41 (−8 to −64)	−56 (−15 to −76)	−59 (−34 to −75)	−74 (−55 to −87)	−73 (−39 to −86)	−81 (−76 to −95)
Serum albumin (g/dl), mean ± SD	2.6 ± 0.3	2.8 ± 0.5	3.1 ± 0.6	3.2 ± 0.5	3.5 ± 0.5	3.4 ± 0.6	3.6 ± 0.5
Complete or partial remission, %	–	7	29	54	56	59	74
Immunological remission, %	–	–	–	20 [77]	–	–	24 [92]
Cumulative dose of GC (g), mean ± SD	–	3.9 ± 0.4	3.9 ± 0.4	7.6 ± 0.9	7.6 ± 0.9	11.3 ± 1.5	11.3 ± 1.5
Cumulative dose of CYC (g), mean ± SD	–	–	3.7 ± 1.1	3.7 ± 1.1	7 ± 2.1	7 ± 2.1	10.2 ± 3.2

A recent provocative retrospective case series described a high rate of complete remission (CR) at 24 months with the combination of low-dose CYC plus RTX and GC [55]. Combinations of CYC and RTX may be particularly attractive in high-risk patients—allowing a minimization of GC doses and a more rapid tapering—and deserve to be prospectively evaluated.

## TIP 5. DO NOT USE GC MONOTHERAPY, MYCOPHENOLATE MOFETIL (MMF) OR IMMUNOSUPPRESSANTS OTHER THAN RTX, CNI OR CYC IN THE INITIAL TREATMENT OF MN

Conflicting results were reported by two controlled trials performed several decades ago testing GC monotherapy in MN: deterioration of glomerular filtration rate was significantly more rapid in placebo-treated than in prednisone-treated patients in one study [56], while in a subsequent study [57], no significant differences were found between prednisone given on alternate days versus no specific treatment. Other observational works have also presented data in favour of and against GC monotherapy [58, 59]. In the more recent STARMEN trial, a significant decrease in proteinuria was observed (Table 2) after the first month of GC therapy, even before starting the CYC cycle. In any case, given the inconsistent results between different studies, the known side effects of GC and the proven efficacy of other therapeutic strategies, the use of GC monotherapy for treating MN should be avoided.

Although a potential beneficial effect of MMF in combination with GC, CNI or other immunosuppressants cannot be dismissed [60, 61], MMF monotherapy did not show superiority as compared with conservative treatment in a prospective study [62]. Currently, and until more conclusive data become available, the use of MMF in MN does not seem advisable.

## TIP 6. DO NOT FORGET AVERTIBLE INFECTIOUS COMPLICATIONS IN PATIENTS RECEIVING IST

Patients with MN receiving immunosuppressive drugs are at increased risk for infections. Although specific recommendations on screening for latent infections and antimicrobial prophylaxis are currently lacking in subjects with MN, general recommendations for patients with glomerular diseases on immunosuppression have been outlined by international guidelines [63]. It is worth noting that much of the evidence regarding this area is extrapolated from other settings, such as patients with rheumatic disease.

Latent tuberculosis infection should be ruled out according to local practice and treatment should be instituted along with IST in positive cases [63]. Immunosuppressive agents (particularly RTX) can induce hepatitis B virus reactivation/exacerbation [64], therefore, it is imperative to perform serologic tests (HBsAg and HBcAb) in all patients and treat accordingly [65]. Other pathogens that should be screened include hepatitis C virus, human immunodeficiency virus, syphilis and *Strongyloides stercoralis* (only in high-risk populations) [63, 66].

*Pneumocystis* prophylaxis should be strongly considered in patients receiving high-dose GC [67], RTX or CYC (Table 3). Current herpes zoster virus (HZV) prophylaxis recommendations are vague [63]. We advise the use of acyclovir or valacyclovir only in selected cases, such as highly immunosuppressed patients with repeated episodes of HZV or those on anti-CD38 antibodies. Hypogammaglobulinaemia should be monitored in RTX-treated patients since it has been associated with a significant increase in severe infections and increased mortality. Immunoglobulin replacement therapy may be of benefit in these cases [68].

**Table 3:** Antimicrobial prophylaxis for patients with MN on immunosuppressive treatment.

Aetiologic agent	Prophylaxis advise	Duration/regimen
*Pneumocystis jirovecii* ^a^		
Prednisone	Start when prednisone dose ≥16 mg/day with foreseen duration >8 weeks	Maintain until prednisone dose <7.5 mg/day
CYC	Contemplate in all patients	Maintain for at least 3 months after last CYC dose
RTX	Contemplate in all patients	Maintain for at least 6 months after last RTX dose
*Mycobacterium tuberculosis*		
	Start treatment for latent tuberculosis once active disease has been ruled out	Isoniazid for 9 months (5 mg/kg/day, maximum 300 mg/day) or rifampicin for 4 months (10 mg/kg/day, maximum 600 mg/day) or isoniazid + rifampicin for 3 months
Hepatitis B virus (HBV)		
	Use of RTX or CYC in patients with HBV infection should be assessed on a case-by-case basis	Sustained virologic response is advised prior to immunosuppression initiation
*Strongyloides stercoralis*		
	Screening in patients who come from areas with a high prevalence of tropical diseases (residents or recent travel history)	Ivermectin 200 μg/kg for 2 consecutive days or 1 week apart

^a^ *Pneumocystis* prophylaxis is based on trimethoprim–sulfamethoxazole [one double-strength (160/800 mg) tablet three times per week. Second-line treatments include dapsone 100 mg once daily, atovaquone 1500 mg once daily and pentamidine 300 mg (nebulized) once monthly.

Vaccination against pneumococcus (heptavalent and 23-valent) and influenza (annually) should be performed in all patients. Prevention of HZV may be considered with the Shingrix vaccine. Recommendations regarding vaccination against severe acute respiratory syndrome coronavirus 2 are frequently amended, hence we advise following local or national guidelines, always considering that patients on IST fall into the high-risk category.

## TIP 7. MONITOR AND PERSONALIZE IST BY CLOSELY MEASURING KIDNEY FUNCTION, PROTEINURIA, SERUM ALBUMIN AND ANTI-PLA2R LEVELS

In patients with anti-PLA2R positivity, following the tendency of antibody titres is highly useful as a guide to evaluate the efficacy of the chosen IST as well as dose and duration [12, 13, 17, 18, 69, 70, 71]. The decrease in anti-PLA2R titres precedes a decrease in proteinuria by several weeks and an interval of 2.7 ± 1.7 months has been observed between immunologic and clinical remission [69]. Patients initially considered resistant to RTX because of high baseline anti-PLA2R titres may reach remission after a second RTX course [72].

Although few centres routinely determine antibodies other than anti-PLA2R (such as THSD7A, NELL-1 and others), this scenario will likely change in the near future. Over the last few years, several antigens other than PLA2R with a clear pathogenic implication in NM have been identified [73]. Their standardized determination will probably allow their monitoring in a similar manner to what happened with anti-PLA2R. On the other hand, the associations of these new antigens with different aetiologies and clinical manifestations (autoimmune disorders, drugs, infections, tumours) will change our diagnostic approach, questioning the traditional separation between primary and secondary MN and offering a more personalized approach to diagnosis and treatment of the disease [73].

Monitoring of proteinuria, serum albumin and kidney function are also crucial, not just in anti-PLA2R-negative cases [74]. Cases with immunologic remission not followed by remission of NS have been described [69] and recent evidence suggests that baseline anti-PLA2R titres do not predict disease progression with such confidence after adjusting for proteinuria and kidney function [75, 76]. A subanalysis of the MENTOR trial (NCT01180036) showed that anti-PLA2R antibody titres combined with serum albumin levels after 3 months of treatment was the best method to evaluate the probability of remission [77]. Serum albumin levels are of great value considering that proteinuria can show fluctuations caused by haemodynamic changes or inaccurate urine collection.

Assessment of proteinuria should always be performed with the same method throughout the clinical course. A 24-h urine collection is the gold standard, but determination of the protein:creatinine ratio (PCR) on an aliquot of an attempted 12- to 24-h urine collection is also acceptable. Avoid evaluation based on the albumin:creatinine ratio or random spot PCR [63].

## TIP 8. TREATMENT OF RESISTANT DISEASE SHOULD BE PERFORMED IN A STEPWISE MANNER

**Figure 3:**
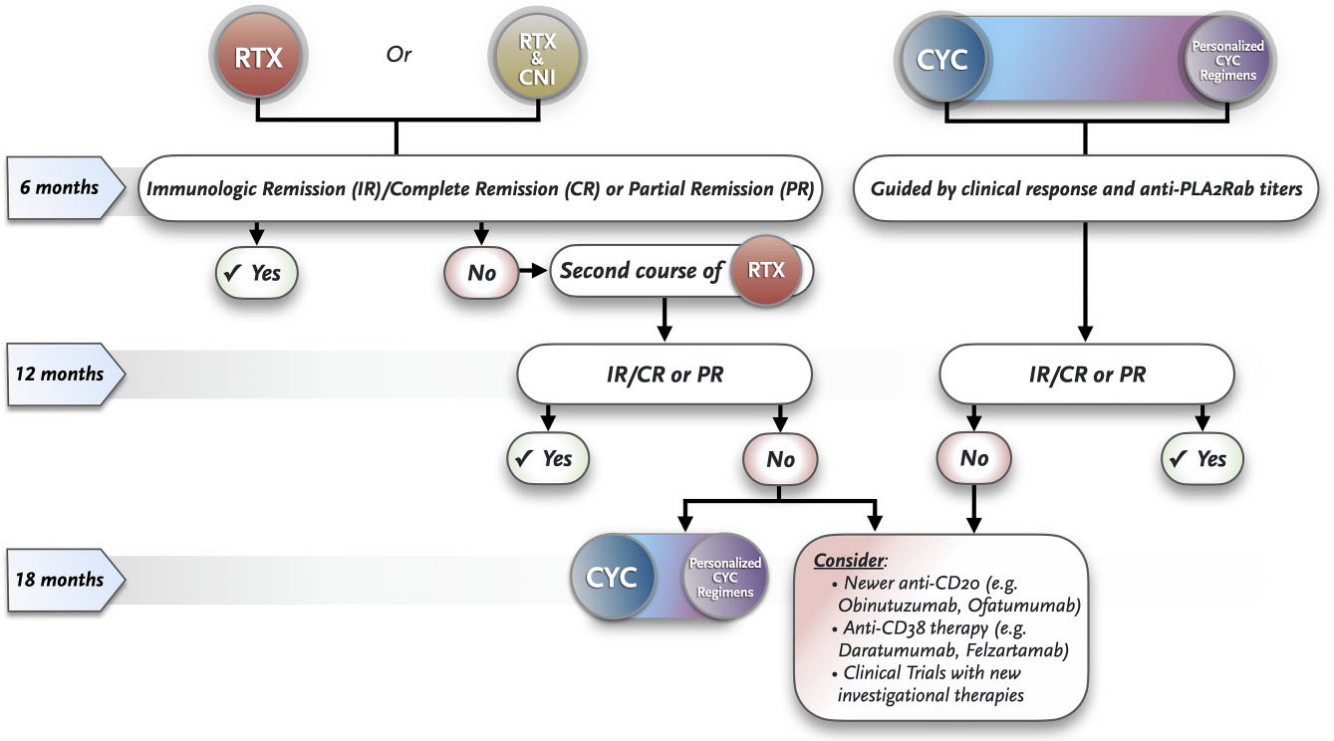
Treatment algorithm according to initial therapeutic regimen.

Although there is no consensus on the definition of resistant MN, it is estimated that ≈30% of patients will not respond to treatment [78]. If no immunologic (persistence of high/unchanged anti-PLA2R antibodies) or clinical (persistent NS/absence of at least PR) improvement is observed after 12 months following RTX or RTX plus CNI, resistant disease should be suspected. In these cases, CYC-based strategies are recommended as the next therapeutic step (Fig. 3), considering that the high efficacy of CYC in MN is supported by solid data [25, 33, 37, 39, 40] (Table 1).

Alternatively, new anti-CD20 monoclonal antibodies may be used in cases of non-response to RTX. Two small case series (*n* = 13 patients) recently reported encouraging results with obinutuzumab in patients with MN refractory to RTX, achieving immunologic remission in most cases and CR or PR in up to 85% of patients [79, 80]. Positive results were also published for ofatumumab in RTX-intolerant or resistant patients (*n* = 17), whereas clinical response (CR or PR) was observed in all cases and immunologic improvement in ≈50% [81]. Obinutuzumab could be a good option in cases refractory or intolerant to RTX, whereas ofatumumab could work in cases with RTX intolerance or in those with anti-RTX antibodies. The latter agents should also be considered in patients without response to GC and CYC.

Non-response to anti-CD20 agents, CNI and CYC can represent a serious medical challenge. In these unfortunate cases, one may consider inclusion in clinical trials with experimental drugs or try anti-CD38 monoclonal antibodies such as daratumumab [82] or proteasome inhibition with bortezomib [83].

## TIP 9. AFTER ATTAINING REMISSION, MONITOR RISK FACTORS FOR RELAPSE AND TREAT RELAPSES BASED ON THE PATIENT'S RISK PROFILE, SIMILAR TO THE FIRST BOUT OF THE DISEASE

The ideal outcome of treating MN with IST is to obtain CR. However, achieving PR is a satisfactory goal given that the risk of kidney failure is much lower than in patients without response [84]. After PR is attained, residual proteinuria can be reduced by means of optimized non-immunosuppressive antiproteinuric treatments previously discussed (tip 1).

The prognosis of PR after IST is similar to that of spontaneous PR, yet a significant proportion of cases (up to 47% in some studies) present with relapses [84]. Relapses are more frequent in patients with PR compared with CR and in cases with persistent low serum albumin (<3.5 g/dl) [85]. Persistence of mild hypoalbuminaemia despite non-nephrotic proteinuria is not uncommon and may indicate an active immunologic state. Beneficial effects of RTX have been described in patients with PR and persistent anti-PLA2R [86], although larger studies are needed.

In contrast, some patients may present with nephrotic-range proteinuria (>3.5 g/24 h) and normal serum albumin (>3.5 g/dl). This subset of patients (nephrotic-range proteinuria without NS) has been well characterized in FSGS [87] but seldom in MN. In our experience, this clinical profile can be observed in patients with PR in whom proteinuria increases to the nephrotic range, mainly due to significant weight gain. Optimization of RAAS blockade and other renoprotective drugs (SGLT2i) together with weight loss usually decreases proteinuria to the non-nephrotic range.

Although there are no studies specifically focused on the treatment of relapses, it seems reasonable to select the type of IST based on risk factors (Fig. 1), similar to the first episode. However, if a new course of CYC-based therapies is considered, it is advisable that the cumulative dose does not exceed 10 g.

## TIP 10. DO NOT FORGET THE UNDERLYING DISEASE THAT LED TO KIDNEY TRANSPLANTATION, AS MN MAY REOCCUR

Primary MN can relapse after kidney transplantation in ≈30–50% of cases [88–90]. Risk factors for recurrent disease include the presence of anti-PLA2R antibodies at the time of kidney transplantation (especially if positivity persists during follow-up), a weak transplant immunosuppressive regimen and an aggressive disease with rapid progression towards renal failure [88, 91, 92].

**Figure 4:**
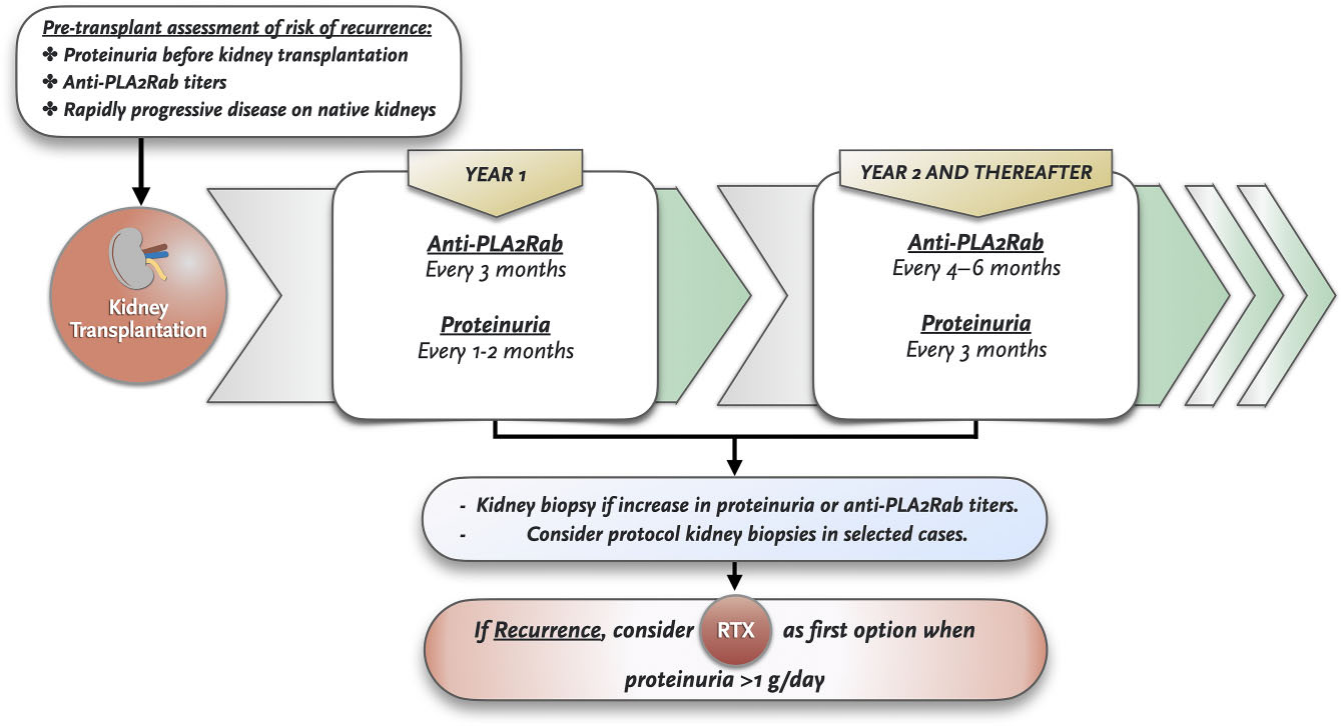
Algorithm for pretransplant risk recurrence of MN and post-transplant follow-up recommendations.

Proteinuria and immunologic monitoring should be performed frequently during the first 12–24 months after kidney transplantation. We suggest measuring 24-h proteinuria every 1–2 months during the first year and every 3 months subsequently, as well as anti-PLA2R determination at least every 3 months during the first year and every 4–6 months afterwards. An allograft biopsy should be indicated without delay in cases with increasing anti-PLA2R titres or worsening proteinuria (Fig. 4).

In contrast to native disease, clinical presentation can be highly variable (positive histology without clinical expression, non-nephrotic proteinuria, NS) and spontaneous remissions are less frequently observed [93, 94]. Different studies have shown that MN recurrence is a risk factor for allograft failure [95–97] and adjuvant IST may be required in some cases. After supportive therapy with antiproteinuric agents is initiated, close observation must be implemented. If proteinuria goes beyond 1 g/24 h, treatment with RTX (Fig. 2) should be considered as first-line therapy [63]. Reported remission rates with RTX are variable, but data from relatively large series suggest that partial or complete remission is observed in ≈70–80% of cases as compared with 30–35% with supportive therapy alone [88, 98]. Data on CYC-based regimens is quite scarce [89], but in case of non-response to RTX, its use could be considered. *De novo* MN (unrelated to native kidney disease) is thought to be a type of alloimmune response since the majority of cases are associated with rejection lesions [99–102], therefore therapy should be assessed on a case-by-case basis.

## Data Availability

No new data were generated or analysed in support of this research.

## References

[1] Hladunewich MA, Troyanov S, Calafati J et al. The natural history of the non-nephrotic membranous nephropathy patient. Clin J Am Soc Nephrol 2009;4:1417–22. 10.2215/CJN.01330209 19661220 PMC2736692

[2] Thompson A, Cattran DC, Blank M et al. Complete and partial remission as surrogate end points in membranous nephropathy. J Am Soc Nephrol 2015;26:2930–7. 10.1681/ASN.2015010091 26078365 PMC4657845

[3] Nuffield Department of Population Health Renal Studies Group, SGLT2 inhibitor Meta-Analysis Cardio-Renal Trialists’ Consortium . Impact of diabetes on the effects of sodium glucose co-transporter-2 inhibitors on kidney outcomes: collaborative meta-analysis of large placebo-controlled trials. Lancet 2022;400:1788–801. 10.1016/S0140-6736(22)02074-8 36351458 PMC7613836

[4] Heerspink HJL, Stefánsson BV, Correa-Rotter R et al. Dapagliflozin in patients with chronic kidney disease. N Engl J Med 2020;383:1436–46. 10.1056/NEJMoa2024816 32970396

[5] EMPA-KIDNEY Collaborative Group, Herrington WG, Staplin N et al. Empagliflozin in patients with chronic kidney disease. N Engl J Med 2023;388:117–27. 36331190 10.1056/NEJMoa2204233 PMC7614055

[6] Rojas-Rivera JE, Ortiz A, Fervenza FC. Novel treatments paradigms: membranous nephropathy. Kidney Int Rep 2023;8:419–31. 10.1016/j.ekir.2022.12.011 36938069 PMC10014375

[7] Caravaca-Fontán F, Stevens K, Padrón M et al. Sodium-glucose cotransporter 2 inhibition in primary and secondary glomerulonephritis. Nephrol Dial Transplant 2024;39:328–40. 37550217 10.1093/ndt/gfad175

[8] Davison AM, Cameron JS, Kerr DN et al. The natural history of renal function in untreated idiopathic membranous glomerulonephritis in adults. Clin Nephrol 1984;22:61–7. 6478673

[9] Schieppati A, Mosconi L, Perna A et al. Prognosis of untreated patients with idiopathic membranous nephropathy. N Engl J Med 1993;329:85–9. 10.1056/NEJM199307083290203 8510707

[10] Polanco N, Gutiérrez E, Covarsí A et al. Spontaneous remission of nephrotic syndrome in idiopathic membranous nephropathy. J Am Soc Nephrol 2010;21:697–704. 10.1681/ASN.2009080861 20110379 PMC2844306

[11] Hofstra JM, Beck LH, Beck DM et al. Anti-phospholipase A2 receptor antibodies correlate with clinical status in idiopathic membranous nephropathy. Clin J Am Soc Nephrol 2011;6:1286–91. 10.2215/CJN.07210810 21474589 PMC3109923

[12] Hoxha E, Thiele I, Zahner G et al. Phospholipase A2 receptor autoantibodies and clinical outcome in patients with primary membranous nephropathy. J Am Soc Nephrol 2014;25:1357–66. 10.1681/ASN.2013040430 24610926 PMC4033365

[13] Ruggenenti P, Debiec H, Ruggiero B et al. Anti-phospholipase A2 receptor antibody titer predicts post-rituximab outcome of membranous nephropathy. J Am Soc Nephrol 2015;26:2545–58. 10.1681/ASN.2014070640 25804280 PMC4587688

[14] Seitz-Polski B, Debiec H, Rousseau A et al. Phospholipase A2 receptor 1 epitope spreading at baseline predicts reduced likelihood of remission of membranous nephropathy. J Am Soc Nephrol 2018;29:401–8. 10.1681/ASN.2017070734 29114041 PMC5791059

[15] Pei Y, Cattran D, Greenwood C. Predicting chronic renal insufficiency in idiopathic membranous glomerulonephritis. Kidney Int 1992;42:960–6. 10.1038/ki.1992.374 1453588

[16] Liang Y, Wan J, Chen Y et al. Serum anti-phospholipase A2 receptor (PLA2R) antibody detected at diagnosis as a predictor for clinical remission in patients with primary membranous nephropathy: a meta-analysis. BMC Nephrol 2019;20:360. 10.1186/s12882-019-1544-2 31533641 PMC6749720

[17] Ronco P, Plaisier E, Debiec H. The role of PLA2R antibody monitoring: what we know and what we do not know. Nephrol Dial Transplant 2023;38:826–33. 10.1093/ndt/gfab356 34910212

[18] van de Logt AE, Fresquet M, Wetzels JF et al. The anti-PLA2R antibody in membranous nephropathy: what we know and what remains a decade after its discovery. Kidney Int 2019;96:1292–302. 10.1016/j.kint.2019.07.014 31611068

[19] Howman A, Chapman TL, Langdon MM et al. Immunosuppression for progressive membranous nephropathy: a UK randomised controlled trial. Lancet 2013;381:744–51. 10.1016/S0140-6736(12)61566-9 23312808 PMC3590447

[20] Torres A, Domínguez-Gil B, Carreño A et al. Conservative versus immunosuppressive treatment of patients with idiopathic membranous nephropathy. Kidney Int 2002;61:219–27. 10.1046/j.1523-1755.2002.00124.x 11786104

[21] Ruggenenti P, Cravedi P, Chianca A et al. Rituximab in idiopathic membranous nephropathy. J Am Soc Nephrol 2012;23:1416–25. 10.1681/ASN.2012020181 22822077 PMC3402291

[22] Bomback AS, Derebail VK, McGregor JG et al. Rituximab therapy for membranous nephropathy: a systematic review. Clin J Am Soc Nephrol 2009;4:734–44. 10.2215/CJN.05231008 19279120 PMC2666426

[23] Dahan K, Debiec H, Plaisier E et al. Rituximab for severe membranous nephropathy: a 6-month trial with extended follow-up. J Am Soc Nephrol 2017;28:348–58. 10.1681/ASN.2016040449 27352623 PMC5198292

[24] Fervenza FC, Appel GB, Barbour SJ et al. Rituximab or cyclosporine in the treatment of membranous nephropathy. N Engl J Med 2019;381:36–46. 10.1056/NEJMoa1814427 31269364

[25] Scolari F, Delbarba E, Santoro D et al. Rituximab or cyclophosphamide in the treatment of membranous nephropathy: the RI-CYCLO randomized trial. J Am Soc Nephrol 2021;32:972–82. 10.1681/ASN.2020071091 33649098 PMC8017548

[26] Cattran DC, Appel GB, Hebert LA et al. Cyclosporine in patients with steroid-resistant membranous nephropathy: a randomized trial. Kidney Int 2001;59:1484–90. 10.1046/j.1523-1755.2001.0590041484.x 11260412

[27] Praga M, Barrio V, Juárez GF et al. Tacrolimus monotherapy in membranous nephropathy: a randomized controlled trial. Kidney Int 2007;71:924–30. 10.1038/sj.ki.5002215 17377504

[28] Caro J, Gutiérrez-Solís E, Rojas-Rivera J et al. Predictors of response and relapse in patients with idiopathic membranous nephropathy treated with tacrolimus. Nephrol Dial Transplant 2015;30:467–74. 10.1093/ndt/gfu306 25274748

[29] Ramachandran R, Hn HK, Kumar V et al. Tacrolimus combined with corticosteroids versus modified Ponticelli regimen in treatment of idiopathic membranous nephropathy: randomized control trial. Nephrology 2016;21:139–46. 10.1111/nep.12569 26205759

[30] Ramachandran R, Kumar V, Bharati J et al. Long-term follow-up of cyclical cyclophosphamide and steroids versus tacrolimus and steroids in primary membranous nephropathy. Kidney Int Rep 2021;6:2653–60. 10.1016/j.ekir.2021.07.028 34622104 PMC8484506

[31] Cattran DC, Alexopoulos E, Heering P et al. Cyclosporin in idiopathic glomerular disease associated with the nephrotic syndrome: workshop recommendations. Kidney Int 2007;72:1429–47. 10.1038/sj.ki.5002553 17898700

[32] Segarra A, Praga M, Ramos N et al. Successful treatment of membranous glomerulonephritis with rituximab in calcineurin inhibitor-dependent patients. Clin J Am Soc Nephrol 2009;4:1083–8. 10.2215/CJN.06041108 19478097 PMC2689890

[33] Fernández-Juárez G, Rojas-Rivera J, van de Logt A-E et al. The STARMEN trial indicates that alternating treatment with corticosteroids and cyclophosphamide is superior to sequential treatment with tacrolimus and rituximab in primary membranous nephropathy. Kidney Int 2021;99:986–98. 10.1016/j.kint.2020.10.014 33166580

[34] Moroni G, Depetri F, Del Vecchio L et al. Low-dose rituximab is poorly effective in patients with primary membranous nephropathy. Nephrol Dial Transplant 2017;32:1691–6. 27387472 10.1093/ndt/gfw251

[35] Waldman M, Beck LH, Braun M et al. Membranous nephropathy: pilot study of a novel regimen combining cyclosporine and rituximab. Kidney Int Rep 2016;1:73–84. 10.1016/j.ekir.2016.05.002 27942609 PMC5138549

[36] Ponticelli C, Zucchelli P, Passerini P et al. A randomized trial of methylprednisolone and chlorambucil in idiopathic membranous nephropathy. N Engl J Med 1989;320:8–13. 10.1056/NEJM198901053200102 2642605

[37] Jha V, Ganguli A, Saha TK et al. A randomized, controlled trial of steroids and cyclophosphamide in adults with nephrotic syndrome caused by idiopathic membranous nephropathy. J Am Soc Nephrol 2007;18:1899–904. 10.1681/ASN.2007020166 17494881

[38] Ponticelli C, Zucchelli P, Passerini P et al. Methylprednisolone plus chlorambucil as compared with methylprednisolone alone for the treatment of idiopathic membranous nephropathy. N Engl J Med 1992;327:599–603. 10.1056/NEJM199208273270904 1640953

[39] Ponticelli C, Passerini P, Altieri P et al. A randomized study comparing methylprednisolone plus chlorambucil versus methylprednisolone plus cyclophosphamide in idiopathic membranous nephropathy. J Am Soc Nephrol 1998;9:444–50. 10.1681/ASN.V93444 9513907

[40] Hofstra JM, Wetzels JFM. Alkylating agents in membranous nephropathy: efficacy proven beyond doubt. Nephrol Dial Transplant 2010;25:1760–6. 10.1093/ndt/gfq017 20133280

[41] Ponticelli C, Zucchelli P, Passerini P et al. A 10-year follow-up of a randomized study with methylprednisolone and chlorambucil in membranous nephropathy. Kidney Int 1995;48:1600–4. 10.1038/ki.1995.453 8544420

[42] van de Logt A-E, Dahan K, Rousseau A et al. Immunological remission in PLA2R-antibody-associated membranous nephropathy: cyclophosphamide versus rituximab. Kidney Int 2018;93:1016–7. 10.1016/j.kint.2017.12.019 29571438

[43] Naik S, Pal D, Shukla S et al. Rituximab in patients with primary membranous nephropathy with high immunologic risk. Kidney Int Rep 2023;8:1660–4. 10.1016/j.ekir.2023.05.009 37547518 PMC10403650

[44] van den Brand JAJG, Ruggenenti P, Chianca A et al. Safety of rituximab compared with steroids and cyclophosphamide for idiopathic membranous nephropathy. J Am Soc Nephrol 2017;28:2729–37. 10.1681/ASN.2016091022 28487395 PMC5576929

[45] van den Brand JAJG, van Dijk PR, Hofstra JM et al. Cancer risk after cyclophosphamide treatment in idiopathic membranous nephropathy. Clin J Am Soc Nephrol 2014;9:1066–73. 10.2215/CJN.08880813 24855280 PMC4046727

[46] Trujillo H, Alonso M, Praga M. New ways of understanding membranous nephropathy. Nephron 2020;144:261–71. 10.1159/000506948 32229730

[47] du Buf-Vereijken PWG, Branten AJW, Wetzels JFM. Cytotoxic therapy for membranous nephropathy and renal insufficiency: improved renal survival but high relapse rate. Nephrol Dial Transplant 2004;19:1142–8. 10.1093/ndt/gfh036 14993502

[48] Eriguchi M, Oka H, Mizobuchi T et al. Long-term outcomes of idiopathic membranous nephropathy in Japanese patients treated with low-dose cyclophosphamide and prednisolone. Nephrol Dial Transplant 2009;24:3082–8. 10.1093/ndt/gfp251 19465558

[49] Dede F, Ayili D, Sahiner S. Effective treatment administration of cyclophosphamide in membranous nephropathy. J Nephrol 2008;21:560–5. 18651546

[50] Kanigicherla DAK, Hamilton P, Czapla K et al. Intravenous pulse cyclophosphamide and steroids induce immunological and clinical remission in New-incident and relapsing primary membranous nephropathy. Nephrology 2018;23:60–68. 10.1111/nep.12955 27778424

[51] Yuan J, Fang W, Zhang W et al. Treatment of nephrotic idiopathic membranous nephropathy with monthly i.v. pulse cyclophosphamide and oral steroids: a single centre's retrospective study. Nephrology 2011;16:440–5. 10.1111/j.1440-1797.2010.01427.x 21091923

[52] Luzardo L, Ottati G, Cabrera J et al. Substitution of oral for intravenous cyclophosphamide in membranous nephropathy. Kidney360 2020;1:943–9. 10.34067/KID.0002802020 35369556 PMC8815588

[53] Mathrani V, Alejmi A, Griffin S et al. Intravenous cyclophosphamide and oral prednisolone is a safe and effective treatment option for idiopathic membranous nephropathy. Clin Kidney J 2017;10:450–4. 10.1093/ckj/sfw152 28852480 PMC5570044

[54] Vink CH, van de Logt A-E, van der Molen RG et al. Antibody-guided therapy in phospholipase A2 receptor-associated membranous nephropathy. Kidney Int Reps 2023;8:432–41. 10.1016/j.ekir.2022.12.003 PMC10014436 36938074

[55] Zonozi R, Laliberte K, Huizenga NR et al. Combination of rituximab, low-dose cyclophosphamide, and prednisone for primary membranous nephropathy: a case series with extended follow up. Am J Kidney Dis 2021;78:793–803. 10.1053/j.ajkd.2021.04.014 34174365

[56] Collaborative Study of the Adult Idiopathic Nephrotic Syndrome . A controlled study of short-term prednisone treatment in adults with membranous nephropathy. N Engl J Med 1979;301:1301–6. 10.1056/NEJM197912133012401 388220

[57] Cattran DC, Delmore T, Roscoe J et al. A randomized controlled trial of prednisone in patients with idiopathic membranous nephropathy. N Engl J Med 1989;320:210–5. 10.1056/NEJM198901263200403 2643046

[58] Alexopoulos E, Sakellariou G, Memmos D et al. Cyclophosphamide provides no additional benefit to steroid therapy in the treatment of idiopathic membranous nephropathy. Am J Kidney Dis 1993;21:497–503. 10.1016/S0272-6386(12)80395-7 8488817

[59] Cameron JS, Healy MJ, Adu D. The Medical Research Council trial of short-term high-dose alternate day prednisolone in idiopathic membranous nephropathy with nephrotic syndrome in adults. The MRC Glomerulonephritis Working Party. Q J Med 1990;74:133–56. 10.1093/oxfordjournals.qjmed.a068422 2189149

[60] Duan Y, Bai Y, Guo W et al. Multitarget therapy with a corticosteroid, cyclosporine and mycophenolate mofetil for idiopathic membranous nephropathy: a prospective randomized controlled trial. Nephrol Dial Transplant 2023;39:95–102. 10.1093/ndt/gfad156 37437905 PMC10730809

[61] Chan TM, Lin AW, Tang SC et al. Prospective controlled study on mycophenolate mofetil and prednisolone in the treatment of membranous nephropathy with nephrotic syndrome. Nephrology 2007;12:576–81. 10.1111/j.1440-1797.2007.00822.x 17995584

[62] Dussol B, Morange S, Burtey S et al. Mycophenolate mofetil monotherapy in membranous nephropathy: A 1-year randomized controlled trial. Am J Kidney Dis 2008;52:699–705. 10.1053/j.ajkd.2008.04.013 18585835

[63] Kidney Disease: Improving Global Outcomes (KDIGO) Glomerular Diseases Work Group . KDIGO 2021 Clinical Practice Guideline for the Management of Glomerular Diseases. Kidney Int 2021;100(4 Suppl):S1–276. 10.1016/j.kint.2021.05.021 34556256

[64] Perrillo RP, Gish R, Falck-Ytter YT. American Gastroenterological Association Institute technical review on prevention and treatment of hepatitis B virus reactivation during immunosuppressive drug therapy. Gastroenterology 2015;148:221–44.e3. 10.1053/j.gastro.2014.10.038 25447852

[65] Berchtold L, Zanetta G, Dahan K et al. Efficacy and safety of rituximab in hepatitis B virus-associated PLA2R-positive membranous nephropathy. Kidney Int Rep 2018;3:486–91. 10.1016/j.ekir.2017.09.009 29725654 PMC5932116

[66] Mejia R, Nutman TB. Screening, prevention, and treatment for hyperinfection syndrome and disseminated infections caused by *Strongyloides stercoralis*. Curr Opin Infect Dis 2012;25:458–63. 10.1097/QCO.0b013e3283551dbd 22691685 PMC3430846

[67] Gupta D, Zachariah A, Roppelt H et al. Prophylactic antibiotic usage for *Pneumocystis jirovecii* pneumonia in patients with systemic lupus erythematosus on cyclophosphamide: a survey of US rheumatologists and the review of literature. J Clin Rheumatol 2008;14:267–72. 10.1097/RHU.0b013e31817a7e30 18679133

[68] Barmettler S, Ong MS, Farmer JR et al. Association of immunoglobulin levels, infectious risk, and mortality with rituximab and hypogammaglobulinemia. JAMA Netw Open 2018;1:e184169. 10.1001/jamanetworkopen.2018.4169 30646343 PMC6324375

[69] Ramachandran R, Yadav AK, Kumar V et al. Temporal association between PLA2R antibodies and clinical outcomes in primary membranous nephropathy. Kidney Int Rep 2018;3:142–7. 10.1016/j.ekir.2017.09.001 29340324 PMC5762964

[70] Bech AP, Hofstra JM, Brenchley PE et al. Association of anti-PLA_2_R antibodies with outcomes after immunosuppressive therapy in idiopathic membranous nephropathy. Clin J Am Soc Nephrol 2014;9:1386–92. 10.2215/CJN.10471013 25035272 PMC4123402

[71] Glassock RJ, Fervenza FC. “Precision” medicine in membranous nephropathy: serology-guided therapy. Kidney Int Rep 2023;8:397–400. 10.1016/j.ekir.2023.01.024 36938066 PMC10014438

[72] Dahan K, Johannet C, Esteve E et al. Retreatment with rituximab for membranous nephropathy with persistently elevated titers of anti-phospholipase A2 receptor antibody. Kidney Int 2019;95:233–4. 10.1016/j.kint.2018.08.045 30606419

[73] Sethi S, Beck LH, Glassock RJ et al. Mayo Clinic consensus report on membranous nephropathy: proposal for a novel classification. Kidney Int 2023;104:1092–102. 10.1016/j.kint.2023.06.032 37795587

[74] Caravaca-Fontán F, Fernández-Juárez GM, Floege J et al. The management of membranous nephropathy-an update. Nephrol Dial Transplant 2022;37:1033–42. 10.1093/ndt/gfab316 34748001

[75] Ragy O, Bate S, Bukhari S et al. PLA2R antibody does not outperform conventional clinical markers in predicting outcomes in membranous nephropathy. Kidney Int Rep 2023;8:1605–15. 10.1016/j.ekir.2023.05.019 37547510 PMC10403689

[76] van de Logt A-E, Justino J, Vink CH et al. Anti-PLA2R1 antibodies as prognostic biomarker in membranous nephropathy. Kidney Int Rep 2021;6:1677–86. 10.1016/j.ekir.2021.04.002 34169209 PMC8207302

[77] Barbour SJ, Fervenza FC, Induruwage D et al. Anti-PLA2R antibody levels and clinical risk factors for treatment nonresponse in membranous nephropathy. Clin J Am Soc Nephrol 2023;18:1283–93. 10.2215/CJN.0000000000000237 37471101 PMC10578640

[78] Caravaca-Fontán F, Yandian F, Fervenza FC. Future landscape for the management of membranous nephropathy. Clin Kidney J 2023;16:1228–38. 10.1093/ckj/sfad041 37529655 PMC10387398

[79] Sethi S, Kumar S, Lim K et al. Obinutuzumab is effective for the treatment of refractory membranous nephropathy. Kidney Int Rep 2020;5:1515–8. 10.1016/j.ekir.2020.06.030 32954076 PMC7486170

[80] Klomjit N, Fervenza FC, Zand L. Successful treatment of patients with refractory PLA2R-associated membranous nephropathy with obinutuzumab: a report of 3 cases. Am J Kidney Dis 2020;76:883–8. 10.1053/j.ajkd.2020.02.444 32311405

[81] Podestà MA, Trillini M, Portalupi V et al. Ofatumumab in rituximab-resistant and rituximab-intolerant patients with primary membranous nephropathy: a case series. Am J Kidney Dis 2024;83:340–9.e1. 10.1053/j.ajkd.2023.08.010 37777061

[82] Vink CH, van Cranenbroek B, van der Heijden JW et al. Daratumumab for multidrug-resistant phospholipase-A2 receptor-related membranous nephropathy. Kidney Int 2022;101:646–7. 10.1016/j.kint.2021.12.019 35190038

[83] Salhi S, Ribes D, Colombat M et al. Bortezomib plus dexamethasone for rituximab-resistant PLA2R^+^ membranous nephropathy. Kidney Int 2021;100:708–9. 10.1016/j.kint.2021.04.011 34420663

[84] Troyanov S, Wall CA, Miller JA et al. Idiopathic membranous nephropathy: definition and relevance of a partial remission. Kidney Int 2004;66:1199–205. 10.1111/j.1523-1755.2004.00873.x 15327418

[85] Lee T, Chung Y, Poulton CJ et al. Serum albumin at partial remission predicts outcomes in membranous nephropathy. Kidney Int Rep 2020;5:706–17. 10.1016/j.ekir.2020.02.1030 32405591 PMC7210705

[86] Georges E, Johanet C, Plaisier E et al. Efficacy of rituximab in a patient with partial clinical remission and persistent circulating PLA2R-Ab. Kidney Int Rep 2019;4:1027–30. 10.1016/j.ekir.2019.03.002 31312775 PMC6609820

[87] Praga M, Morales E, Herrero JC et al. Absence of hypoalbuminemia despite massive proteinuria in focal segmental glomerulosclerosis secondary to hyperfiltration. Am J Kidney Dis 1999;33:52–8. 10.1016/S0272-6386(99)70257-X 9915267

[88] Hullekes F, Uffing A, Verhoeff R et al. Recurrence of membranous nephropathy after kidney transplantation: a multicenter retrospective cohort study. Am J Transplant 2024;doi: 10.1016/j.ajt.2024.01.036. 10.1016/j.ajt.2024.01.036 38341027

[89] Sprangers B, Lefkowitz GI, Cohen SD et al. Beneficial effect of rituximab in the treatment of recurrent idiopathic membranous nephropathy after kidney transplantation. Clin J Am Soc Nephrol 2010;5:790–7. 10.2215/CJN.04120609 20185599 PMC2863969

[90] Cosio FG, Cattran DC. Recent advances in our understanding of recurrent primary glomerulonephritis after kidney transplantation. Kidney Int 2017;91:304–14. 10.1016/j.kint.2016.08.030 27837947

[91] Kattah A, Ayalon R, Beck LH et al. Anti-phospholipase A_2_ receptor antibodies in recurrent membranous nephropathy. Am J Transplant 2015;15:1349–59. 10.1111/ajt.13133 25766759 PMC4472303

[92] Seitz-Polski B, Payré C, Ambrosetti D et al. Prediction of membranous nephropathy recurrence after transplantation by monitoring of anti-PLA2R1 (M-type phospholipase A2 receptor) autoantibodies: a case series of 15 patients. Nephrol Dial Transplant 2014;29:2334–42. 10.1093/ndt/gfu252 25063424

[93] Leon J, Pérez-Sáez MJ, Batal I et al. Membranous nephropathy posttransplantation: an update of the pathophysiology and management. Transplantation 2019;103:1990–2002. 10.1097/TP.0000000000002758 31568231

[94] Dabade TS, Grande JP, Norby SM et al. Recurrent idiopathic membranous nephropathy after kidney transplantation: a surveillance biopsy study. Am J Transplant 2008;8:1318–22. 10.1111/j.1600-6143.2008.02237.x 18444918

[95] Allen PJ, Chadban SJ, Craig JC et al. Recurrent glomerulonephritis after kidney transplantation: risk factors and allograft outcomes. Kidney Int 2017;92:461–9. 10.1016/j.kint.2017.03.015 28601198

[96] Pippias M, Stel VS, Aresté-Fosalba N et al. Long-term kidney transplant outcomes in primary glomerulonephritis: analysis from the ERA-EDTA Registry. Transplantation 2016;100:1955–62. 10.1097/TP.0000000000000962 26588008

[97] Pruthi R, McClure M, Casula A et al. Long-term graft outcomes and patient survival are lower posttransplant in patients with a primary renal diagnosis of glomerulonephritis. Kidney Int 2016;89:918–26. 10.1016/j.kint.2015.11.022 26924061

[98] Grupper A, Cornell LD, Fervenza FC et al. Recurrent membranous nephropathy after kidney transplantation: treatment and long-term implications. Transplantation 2016;100:2710–6. 10.1097/TP.0000000000001056 26720301

[99] Schwarz A, Krause PH, Offermann G et al. Impact of de novo membranous glomerulonephritis on the clinical course after kidney transplantation. Transplantation 1994;58:650–4. 10.1097/00007890-199409270-00002 7940683

[100] Truong L, Gelfand J, D'Agati V et al. De novo membranous glomerulonephropathy in renal allografts: a report of ten cases and review of the literature. Am J Kidney Dis 1989;14:131–44. 10.1016/S0272-6386(89)80189-1 2667346

[101] Wen J, Xie K, Zhang M et al. HLA-DR, and not PLA2R, is expressed on the podocytes in kidney allografts in de novo membranous nephropathy. Medicine (Baltimore) 2016;95:e4809. 10.1097/MD.0000000000004809 27631233 PMC5402576

[102] Monga G, Mazzucco G, Basolo B et al. Membranous glomerulonephritis (MGN) in transplanted kidneys: morphologic investigation on 256 renal allografts. Mod Pathol 1993;6:249–58. 8346172

